# The clinical impact of direct-acting antiviral treatment on patients affected by hepatitis C virus-related oral lichen planus: a cohort study

**DOI:** 10.1007/s00784-022-04507-9

**Published:** 2022-04-27

**Authors:** Dario Di Stasio, Alberta Lucchese, Antonio Romano, Luigi Elio Adinolfi, Rosario Serpico, Aldo Marrone

**Affiliations:** 1 Multidisciplinary Department of Medical and Dental Specialties, University of Campania- “Luigi Vanvitelli”, Via L. De Crecchio, 6, 80138 Naples, Italy; 2 Department of Advanced Medical and Surgical Sciences, University of Campania Luigi Vanvitelli, Piazza Miraglia, Naples, Italy

**Keywords:** Oral lichen planus, Hepatitis C infection, Direct-acting antivirals (DAAs), HCV-related OLP

## Abstract

**Objectives:**

Oral lichen planus (OLP) is a chronic inflammatory mucocutaneous disease. Literature supports an association between OLP and Hepatitis C virus (HCV) infection. The current treatment for HCV infection with direct-acting antivirals (DAAs) is highly effective and safe. The aim of this study is to evaluate the clinical impact of viral eradication with DAAs in patients with HCV and OLP.

**Materials and methods:**

For this cohort observational study, 18 patients with HCV and OLP were recruited; all patients received DAAs. Nineteen patients with OLP without HCV were recruited as controls. Both groups received an oral clinical examination, taking photographs of the oral mucosa, at three time points. Size and type of lesions, clinical and efficacy scores, were evaluated at each time point with ImageJ software. Changes were assessed by a general linear model repeated measures analysis. Kruskal–Wallis H and Mann–Whitney U tests were used to evaluate the differences between subgroups.

**Results:**

All patients of the study group reached a sustained virological response. The study group showed a correlation between viral load and clinical status (p < 0.05), higher clinical scores at baseline (p = 0.001) and higher efficacy index than controls (p < 0.001), improving over time (p < 0.001); controls did not show significant changes (p = 0.196). One patient of the experimental group developed oral squamous cell carcinoma (OSCC) of the tongue during the DAAs treatment.

**Conclusions:**

In this study, patients with HCV and OLP showed a worst clinical oral status than controls at baseline. However, treatment for virus eradication can improve the oral lichen planus clinical course.

**Clinical relevance:**

HCV eradication can improve the clinical course of patients with HCV-related OLP.

## Introduction

Hepatitis C virus (HCV) infection is a significant public health problem worldwide. The global prevalence of the infection is about 1%, corresponding to approximately 71.1 million chronically infected people, and 1.75 million new cases are estimated worldwide [1, 2]. Recent data estimated 12 million people with chronic hepatitis C and about 64 000 deaths per year in Europe (WHO European Region) [3]. In the Italian population, the prevalence of HCV-seropositivity is estimated to be around 2%, with a peak incidence among people aged between 55 and 64 years and a higher prevalence in Southern Italy and Insular areas (7.3%) [4, 5]. HCV is widely regarded as a potential oncogenic agent. HCV infection is one of the most common causes of hepatocellular carcinoma, and it has also been associated with non-hepatocellular neoplasms and hematologic malignancies [6–8].

HCV acute infection, often asymptomatic, turns chronic in 60–80% of cases and can evolve toward cirrhosis and hepatocellular carcinoma [9].

Chronic disease is characterized by a broad spectrum of hepatic and extrahepatic clinical manifestations mixed cryoglobulinemia, lymphoproliferative disorders, atherosclerosis, neurological and psychiatric disorders, rheumatological diseases, porphyria cutanea tarda and Sjogren Syndrome [10–13]. OLP is an inflammatory disease characterized by a cell-mediated autoimmune reaction in which lesions result from T CD8 + lymphocytes-induced apoptosis of the epithelial basal cells [14]. This condition is frequently reported in patients with HCV-related chronic hepatitis [15]. Epidemiological evidence supports the association between OLP and HCV infection [16]. A hypothesis of correlation claims that the virus, as it would replicate in the oral mucosa, may attract HCV-specific T lymphocytes [16, 17].

The advent of direct antiviral agents (DAAs) has led to viral eradication in up to 99% of treated patients. Recent studies show that HCV clearance achieved with DAAs improved both liver disease and associated extrahepatic clinical manifestations [18–21].

The aim of this cohort study is to analyze the effect of DAAs therapy on a group of HCV-OLP patients comparing it with a cohort of patients with OLP without HCV infection in a long-term follow-up.

## Methods

For this cohort study, the manuscript was prepared and revised according to the STROBE Statement—checklist of items.

### Patients

Eighteen patients of the study group have been recruited consecutively (from January to March 2018) from the Oral Medicine Division and Internal Medicine Units of the University of Campania "Luigi Vanvitelli." For the study group, patients of both sexes aged over 18 with clinical and histological diagnosis of OLP and a confirmed diagnosis of chronic HCV infection have been enrolled; selected patients were eligible for treatment with DAAs. Nineteen patients of both sexes aged over 18 clinical with histological diagnosis of OLP and tested negatively for HCV infection were consecutively recruited as controls (non-HCV-OLP).

In both groups, patients with oral lesions of different etiopathogenesis or neoplastic types and patients with a positive history of chronic non-viral liver diseases were excluded. Patients with drug‐induced or contact lichenoid reactions, pregnant or lactating women were excluded.

Written informed consent was obtained from all participants. The study was approved by the ethical committee of the University of Campania "Luigi Vanvitelli" (#0015388/i). Details regarding patients of both groups are reported in Table 1. The liver characteristics of the patients in the study group and the class of antiviral drugs administered are summarized in Table 2.

**Table 1:** Demographic data and comorbidities of patients of both groups

	HCV-OLP (18 subjects)	non-HCV-OLP (19 subjects)	P value (2-tailed)
Age	73.00 ± 10.1	70.84 ± 11,22	*p* = 0.543*
Gender (F/M)	13/5	11/8	*p* = 0.362**
Smokers	2	6	*p* = 0.311**
Hypertension disease	12	9	*p* = 0.325**
Kidney disease	4	-	*p* = 0.046**
Diabetes	6	3	*p* = 0.214**
ANA positivity	3	5	*p* = 0.476**
Mixed OLP	11	12	*p* > 0.05**
Erosive OLP	7	7	*p* > 0.05**

^*^Independent sample t-test; **Chi-Square test

**Table 2:** Characteristic of HCV-OLP group. Viral load is expressed in log UI/ml; Clinical severity index (CSI) at the three time points has been reported, as well as the final efficacy index (EI). One patient developed oral squamous cells carcinoma (OSCC) of the tongue and were excluded from subsequent evaluations (t_1_ and t_2_)

Patient	HCV genotype	Viral load (log IU/ml)	Liver status	Previous IFN/treatment	DAAs	OLP form	CSI t_0_	CSI t_1_	CSI t_2_	EI
1	2a/2c	6,8	Cirrhosis	No	Sofo + Riba	Erosive	20,5	16,5	10	2
2	2a/2c	6,9	CH	No	Sofo + Velpa	Mixed	12,5	7	7	2
3	1b	6,8	CH	Yes	Ombi/ Pari/ Rito + Dasa	Erosive	26,5	18,5	15,5	2
4	1b	6,8	CH	No	Sofo + Ledi	Erosive	25,5	10	0	4
5	1b	6,3	Cirrhosis	No	Sofo + Ledi	Mixed	9	5,5	0	4
6	1b	6,9	Cirrhosis	No	Sofo + Ledi	Erosive	34	16	13	3
7	1b	4,9	CH	No	Elba + Grazo	Mixed	8,5	5,5	0	4
8	2a/2c	7,7	CH	No	Gleca + Pibre	Mixed	18,5	10,5	10,5	2
9	2a/2c	6,6	CH	No	Sofo + Velpa	Mixed	28,5	15	13,5	2
10	1b	6,6	CH	NO	Gleca + Pibre	Mixed	5,5	4	4	3
11	1b	6,8	CH	Yes	Elba + Grazo	Mixed	10,5	tongue OSCC		
12	2a/2c	6,5	Cirrhosis	Yes	Sofo + Riba	Erosive	8,5	0	0	4
13	1b	6,4	CH	Yes	Sofo + Velpa	Mixed	14,5	4	0	4
14	3a	6,8	CH	No	Sofo + Velpa	Mixed	4	0	0	4
15	1b	6,82	CH	No	Sofo + Ledi	Mixed	18,5	10	7	3
16	1b	6,14	CH	No	Elba + Grazo	Erosive	11,5	**10**	5,5	3
17	2a/2c	6,5	CH	No	Gleca + Pibre	Mixed	10	4	4	3
18	1b	6,81	CH	Yes	Sofo + Ledi	Erosive	9,5	7,5	4	3

*CSI: Clinical Severity Index; EI: Efficacy Index; CH: Chronic Hepatitis; OSCC: Oral Squamous Cell Caricnoma; Sofo: Sofosbuvir; Riba: Ribavirin; Velpa: Velpatasvir; Ombi: ombitasvir; Pari: Paritaprevir; Rito: Ritonavir; Dasa: Dasabuvir; Ledi: Ledipasvir;*

### Clinical and virological evaluation

All patients received a clinical examination of the oral mucosa and underwent specific serological and clinical tests. Diabetes, hypertension, autoimmune diseases, kidney diseases, and cigarette consumption have been investigated.

Antiviral therapy was prescribed according to European Association for the Study of the Liver (EASL) recommendations [22] and the Italian drug agency (AIFA) eligibility criteria, HCV genotype, grade of liver fibrosis, and prior treatment experience.

The same group was monitored at baseline, during, and after treatment for liver function and antiviral response.

Patients received a complete hepatologic assessment at baseline. Biochemical and hematological parameters were monitored before, during, and after antiviral treatment. Patients were tested for ANA (anti-nuclear antibodies). Hepatic ultrasound and hepatic fibrosis evaluation by Fibroscan [23] were performed at baseline, at the end of treatment, and six months later. Serum HCV-RNA was determined at baseline, at the end of antiviral therapy, and 12 and 24 weeks after stopping treatment to establish whether SVR12 had been achieved. Five patients were non-responsive to or relapsed after previous pegylated-IFN-α + RBV combination therapy, whereas the remaining 13 patients were treatment-naïve.

### Outcomes

The primary outcomes were the sign and size of OLP lesions, which were assessed on the baseline (t_0_), which coincided with the start of antiviral therapy. The follow-up was set at t_1_, 8 weeks after the end of the DAAs therapy and at t_2_ to the last control performed. For the control group, outcomes were assessed at the same time interval, setting the t_1_ four months from t_0_ and the t_2_ to the last examination. All patients in both groups were already undergoing topical therapy for OLP with corticosteroids (clobetasol propionate) at the time of the recruitment, as per guidelines. After the first visit, patients of both groups were periodically followed by an oral medicine expert who modulated the therapy at each visit.

Oral mucosa was photographed to evaluate the lesion area (cm^2^) and its evolution from t_0_ to t_2_. Pictures were performed with a NIKON D5200 digital camera.

Image analysis was performed using Image J 1.52 software [24] to obtain the mucosal areas affected by OLP at t_0_, t_1_, and t_2_. The margin of each lesion was drawn manually within the software tracing function. If more than one lesion were present, each lesion was outlined individually. Two different operators who were blind to the group performed computerized analysis, calculating means and standard deviations. The final assessment has been done by evaluating and comparing the results in t_0_, t_1_, and t_2_. Intraclass correlation (ICC) was calculated to assess the inter-rater reliability.

Clinical severity index (CSI) [25] was assessed to compare the clinical severity of OLP within and between patients and monitor the disease's progression.

The total area of the OLP lesions (TA), the area of the erosive lesions (EA), and, if present, the area of the ulcerated lesions (UA) have been calculated as follows: a) Reticular/hyperkeratotic areas were scored from 0 to 1 (0 = no white striations, 1 = white striations or keratotic papules); b) erosive/erythematous areas were scored from 0 to 3 by area of interest (0 = no lesion, 1 = lesions < 1 cm^2^, 2 = 1 to 3 cm^2^, 3 = lesions > 3 cm^2^); c) ulcerations were scored from 0 to 3 by area of interest (0 = no lesion, 1 = lesions < 1 cm^2^, 2 = 1 to 3 cm^2^, 3 = lesions > 3 cm^2^).The final score was computed by summation of all clinical scores: ***reticular score* + *∑ (erosive score* × *1.5)* + *∑ (ulcerative score* × *2.0)***.

The efficacy index (EI) [26] was calculated for the evaluation of the dimensional change of the lesions at the different time points as follows: [***(size2 − size0)∕ size0]100***; size 0 is the total dimension of the lesions affecting the oral mucosa at the baseline while size 2 is the dimension of the lesions at the last follow-up. The EI was interpreted in this way: complete healing for EI 100%, significant improvement for 75% < EI < 100%, moderate improvement for 25% < EI < 75%, mild improvement for 25% < EI < 0, and no improvement for EI 0%.

### Data analysis and statistics

Statistical analysis within the study subjects was carried out as well as a comparison with the control group. Analyses were performed using R (ver. 4.1.0). 

To assess the statistical power and significance of CSI variation in both groups, power analysis was performed using G*Power 3.1.9.6 (Heinrich Heine University, Düsseldorf, Germany) with a two-tailed repeated measures test, assuming a medium Cohen’s effect size of 0.5 (**α** = 0.05). Sensibility (statistical power) was fixed at 0.80.

Dichotomous variables (gender, smoking habits, hypertension disease, kidney disease, oral findings, diabetes, autoimmune diseases) were analyzed by crosstabs and χ2 test. HCV RNA serum levels were expressed in log IU/ml. Kruskal–Wallis H test was run to determine if there were differences in CSI and clinical scores at t_0_ between the different HCV genotypes; Mann–Whitney U test was used to assess if parameters differed depending on the liver status (Cirrhosis or Chronic Hepatitis).

The statistical significance of continuous variable changes across the study was assessed by a general linear model (GLM) repeated measures analysis. Three factors (time points) and four categorical variables (EA, UA, TA, and CSI) were considered.

Except when otherwise indicated, values are expressed as means (standard deviations) and medians.

Correlation significance was calculated with the Spearman equation. All tests were 2-tailed with a significance level of 0.05. Bonferroni correction has been applied for the analysis of the repeated variables, and the p-value acceptance was set at p = 0.017. A Mann–Whitney U test was run to determine differences in the EI score between the two groups.

## Results

Thirty-seven patients (18 of the HCV-OLP group and 19 non-HCV-OLP) with OLP lesions were enrolled in this study. The mean age was 73.00 (± 10.1) years (ranging from 44 to 87, with a male/female ratio of 5/13) for the study group and 70.84 (± 11.2) (range 43–87 – M/F = 8/11) for the control group. The smoking habits and comorbidities of both groups are listed in Table 1.

At t_0_, eleven patients of the study group presented a mixed form of OLP while seven patients presented a prevalence of erosive lesions; seven patients of the control group showed atrophic-erosive OLP and 12 a mixed form. The oral mucosa of the patients of both groups had white reticular lesions and striae regardless of the form of OLP. The median follow-up was 16 weeks at t_1_ (16–22 weeks) and 92 weeks at t_2_ (92–96 weeks).

### Main characteristics of HCV-OLP patients

Eleven patients were diagnosed with chronic hepatitis and 4 with cirrhosis. All patients of the HCV-OLP group were highly viremic with a median HCV RNA level of 6.79 (range 4.89 to 7.67 log IU/ml. Table 2 shows the main clinical and virological characteristics of the study group.

Unfortunately, one patient of the study group developed oral squamous cell carcinoma (OSCC) of the tongue during the DAAs treatment (elbasvir/grazoprevir) and was excluded from subsequent analysis (t_1_ and t_2_). All patients cleared HCV RNA after DAAs and presented SVR12. No adverse events were reported. Before DAAs, eleven patients had F0-F2 grade of liver fibrosis while 7 had F3-F4. After the treatment, seven patients (38.9%) improved liver fibrosis: three patients improved from F2 to F1, two from F1 to F0, one from F3 to F2, and one patient from F4 to F3.

### Clinical severity index

Mean CSI, EA, UA, and TA (cm^2^) at the different time points were reported in Table 3. The ICC was very high (0.967, p > 0.001), showing a good agreement between the assessors.

**Table 3:** The mean values of Clinical scores in the three time-points of observation within groups. Data have been reported as mean (standard deviation). The mean differences (standard error) between t0 and t1, t1 and t2, and t0 and t2 with p-values have been reported

*HCV-OLP*	*t0*	*t1*	*t2*	*t0-t1*	*t1-t2*	*t0-t2*
*CSI*	15.33 (± 8.62)	8.47 (± 5.59)	5.53 (± 5.35)	6.86 (± 1.05) *p* < *0.001*	2.94 (± 0.73) *p* = *0.001*	9.80 (± 1.16) *p* < *0.001*
*EA (cm*^*2*^*)*	7.52 (± 6.34)	3.33 (± 2.49)	1.86 (± 2.25)	4.19 (± 0.95) *p* < *0.001*	1.47 (± 0.59) *p* = *0.053*	5.66 (± 0.90) *p* < *0.001*
*UA (cm*^*2*^*)*	0.75 (± 1.19)	3.48 (± 2.62)	0.12 (± 0.24)	-2.73 (± 0.44) *p* < *0.001*	0.37 (± 0.44) *p* < *0.001*	0.63 (± 0,19) *p* = *0.007*
*TA(cm*^*2*^*)*	8.27 (± 6.86)	6.81 (± 5.11)	1.98 (± 2.42)	1.46 (± 0.90) *p* = *0.162*	4.83 (± 0.80) *p* < *0.001*	6.29 (± 0.97) *p* < *0.001*
*non HCV-OLP*						
*CSI*	7.79 (± 3.16)	7.97 (± 3.28)	6.68 (± 2.92)	-0.18 (± 0.98) *p* = *1.00*	1.29 (± 0.69) *p* = *0.208*	1.11 (± 1.10) *p* = *0.969*
*EA (cm*^*2*^*)*	3.03 (± 1.98)	3.46 (± 3.06)	2.30 (± 1.91)	-0.43 (± 0.90) *p* = *1.00*	1.16 (± 0.56) *p* = *0.131*	0.74 (± 0.83) *p* = *1.00*
*UA (cm*^*2*^*)*	0.16 (± 0.35)	0.25 (± 0.59)	0.20 (± 0.41)	-0.09 (± 0.42) *p* = *1.00*	0.05 (± 0.41) *p* = *1.00*	-0.04 (± 0.19) *p* = *1.00*
*TA (cm*^*2*^*)*	3.19 (± 1.98)	3.72 (± 3.25)	2.50 (± 2.11)	-0.53 (± 0.85) *p* = *1.00*	1.22 (± 0.76) *p* = *0.358*	0.68 (± 0.91) *p* = *1.00*

*CSI: Clinical Severity Index; EA: Erosive areas; UA: Ulcerative areas; TA. Total affected area*

**Fig. 1:**
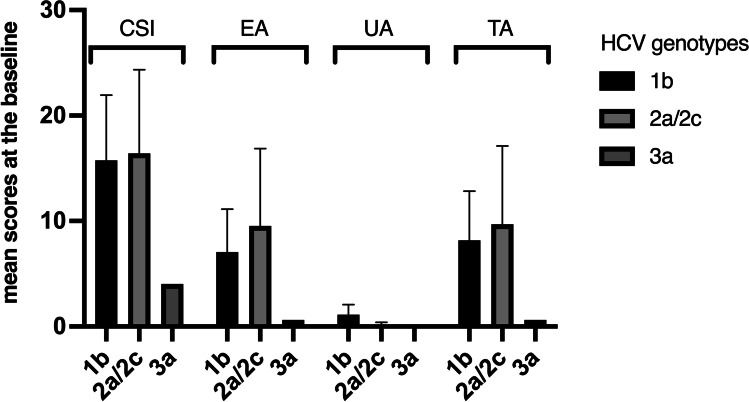
Clinical scores for different genotypes. Error bars (C.I. 95%) are indicated. The distribution was not significantly different in the three subgroups (p > 0.05)

As regards the HCV-OLP group, CSI (*p* = 0.242), EA(*p* = 0.136), UA (*p* = 0.148), and TA (*p* = 0.196) scores at t_0_ were similar for all HCV genotypes (Fig. 1). Stages of liver diseases (Cirrhosis or chronic hepatitis) didn’t correlate with the severity of the oral clinical status (*p* = 0.799).

**Fig. 2:**
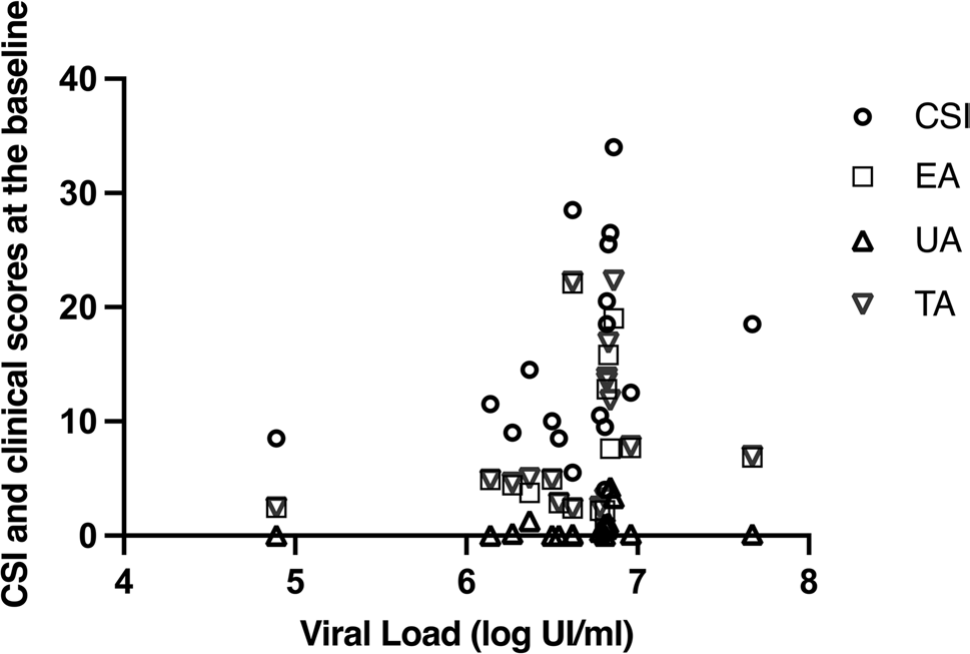
Correlation between clinical scores and viral load (log UI/ml). CSI showed a positive correlation as well as ulcerative areas (UA) and total areas (TA). The erosive area (EA) does not correlates with the viral load

Spearman’s analysis (rs) showed at baseline a positive correlation between HCV viral load (log UI/ml), CSI (rs = 0.558, *p* = 0.016) and UA (rs = 0.478, *p* = 0.045), and TA (rs = 0.534, *p* = 0.023); EA didn’t correlate (rs = 0.463, *p* = 0.053) as shown in Fig. 2. No correlation between smoking habits and comorbidities in both groups has been found. The different viral genotypes didn’t show any correlation with the severity of the oral condition (*p* = 0.686).

The main effect of time on CSI, EA, UA, and TA scores was significant (*p* < 0.001) with two-way interaction between groups in the three time points (*p* < 0.001). Significant differences between groups have been present at the baseline for the CSI (*p* = 0.001), EA (*p* = 0.004), and TA (*p* = 0.012).

**Fig. 3:**
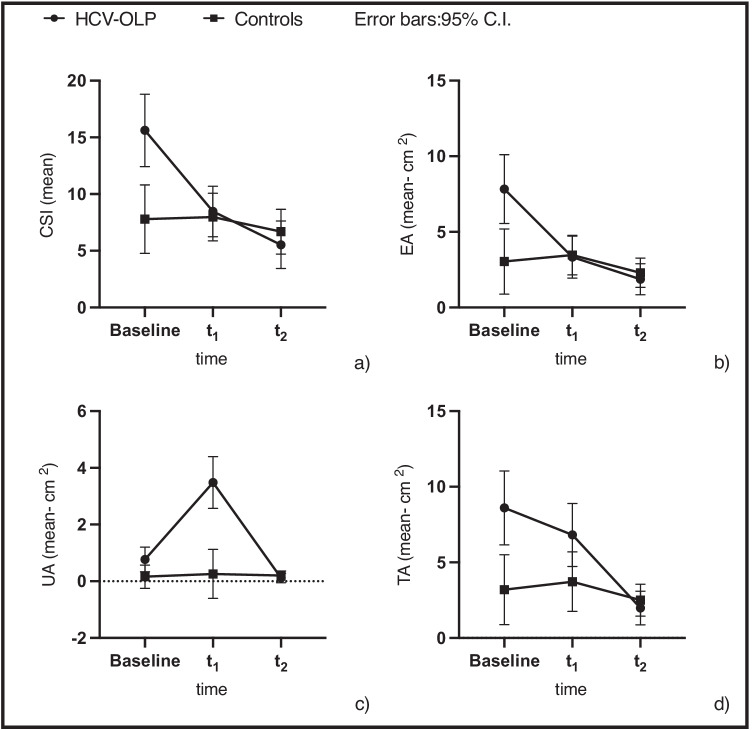
Clinical scores and two-way interaction over time. **a**) Clinical Severity Index (CSI), **b**) erosive area (EA), **c**) ulcerative area and **d**) total area (TA) mean scores at the different time points of observation (including the error bars—95.0% C.I.). Straight line for the HCV-OLP group, dashed line for non-HCV-OLP

At the mid-point, there were no differences for CSI (*p* = 0.744), EA (*p* = 0.89) and TA (*p* = 0.059), as well as at the last follow-up (CSI, *p* = 0.421; EA, *p* = 0.054; TA, *p* = 0.425) as reported in Fig. 3. Regarding the UA, the HCV-OLP group showed an increase in the erosive area in t_1_ after DAAs (*p* < 0.001, Fig. 3c), dropping below the levels of the control group at the last follow-up; however, all patients of the HCV-OLP group clinically improved over time from t_0_ to t_2_ (*p* < 0.001; UA *p* = 0.007).

Significant positive changes have been registered already in t_1_ for the CSI and the EA scores (*p* < 0.001, Fig. 3a,b). non-HCV-OLP group did not show a significant change over time (Table 3).

### Efficacy index (EI)

The median EI in the study group was 3.00 (2.00–4.00). Five patients showed moderate improvement, six patients had significant improvement, and six entirely healed at the last follow-up. In the study group, the median EI was 2.00 (0.00–3.00). Moreover, four patients did not report any improvement, 4 had mild improvement, and 9 showed a moderate improvement; only two patients improved significantly, and no one clinically healed at t_2_. EI scores for patients with HCV-OLP treated with DAAs were significantly higher than controls (*p* < 0.001).

## Discussion

The introduction of the new DAAs drugs for the treatment of HCV infection provides the unprecedented possibility of reaching SVR12 in more than > 90% of the chronically infected patients, with the desired perspective of viral eradication and the consequent clinical advantages in patients with extra-hepatic manifestations of HCV infection such as OLP [21, 27, 28].

In this work, a single-center cohort study analyzing the effect of new DAAs in patients with OLP and HCV infection has been described. This study aimed to analyze the clinical status of the two groups in three time points, using a computerized analysis that allowed to obtain the erosive, ulcerated, and total areas of the mucosa affected by OLP lesions and, consequently, assessing a severity score (CSI) of the disease. This method has been used in other studies on OLP [25, 26, 29].

The first result to be emphasized is that all the above parameters are higher in OLP-HCV patients at baseline. It means that the clinical status of the patients analyzed was worse at t_0_, in patients with HCV infection, presenting more erosive features than the non-HCV-OLP group. Our data are in line with some studies which reported that HCV infection had been more frequently associated with erosive than non-erosive OLP type [30]. After patients of the OLP-HCV group completed the DAAs treatment (t_1_ and t_2_), the clinical course of the disease was comparably aligned with the non-HCV-OLP group with no significant differences in terms of CSI and erosive areas (Fig. 3a,b): this strengthens the hypothesis of the worsening role of HCV infection on the clinical course of patients with OLP. Curiously, the ulcerative lesions of the mucosa increased (UA, Fig. 3c) at t_1_ for the study group and then considerably dropped in t_2_. In a case report, exacerbation of OLP and development of new cutaneous and genital LP upon achieving SVR12 to ledipasvir-sofosbuvir has been reported [31]. The reason for this temporary worsening is unclear, and it needs to be clarified by further studies. Furthermore, it is interesting to note that, the intraoral clinical manifestations at baseline were not correlated with the liver status (*p* = 0.799) and the viral genotype (*p* = 0.686). In contrast, the viral load was positively correlated with the OLP status. As a matter of fact, CSI (rs = 0.558, *p* = 0.016), the presence of ulcerative lesions (rs = 0.478, *p* = 0.045) and the total mucosa affected (rs = 0.534, *p* = 0.023) showed a positive correlation with viraemia levels. In the correlation analysis between erosive areas and viral load, the p-value was slightly higher than the 0.05 (*p* = 0.053—95% CI); a larger sample of patients may be needed to determine this correlation. However, the link between HCV viral load and OLP is still under investigation [32].

Moreover, already at t_1_ (post-eradication), four patients no longer showed oral OLP lesions in the study group, remaining stable even at t_2_. At the last follow-up, in fact, 6/17 patients presented mucosal healing. On the other hand, the control group remains stable over time with a slight improvement due to the clinical and pharmacological management of the disease, but without showing cases of healing.

The data presented are in line with those reported by Nagao et al. [33] who showed clinical improvement in OLP-HCV patients once the virus was eradicated but without a control group and without establishing clinical reference parameters.

In conclusion, antiviral eradication therapy completely changed the prognosis of HCV patients. For patients with HCV-OLP, DAAs therapy appears to be promising as it can improve both the liver disease and extra-hepatic manifestations OLP-related. Nevertheless, the current research on the effects of DAA treatment on OLP in patients with HCV is limited. With these premises, we hypothesized and evaluated whether DAAs also influenced HCV-OLP. Once the virus has been eradicated, indeed, the two groups showed an overlapping trend, giving the possibility to hypothesize that HCV acts as a pathogenic cofactor that is added to the inflammatory processes underlying the etiology of OLP. The data presented confirm this hypothesis and lay the foundations for further studies, including a larger sample size and comparative histological and serological analysis. The limitation of this study is that for evaluating the endpoints, it would have been correct to select a group of patients affected by HCV-OLP not exposed to eradication therapy. However, given the excellent results in terms of SVR12 and safety of treatment with DAAs, and the European Union's commitment to eradicating the virus, authors considered it not possible in ethical terms. Given the availability of effective antiviral therapies, if further studies confirm these data, it could be necessary that people with a clinically severe OLP should be routinely tested for hepatitis C.

Although a causative role of chronic HCV infection on OLP occurrence is not yet definitively demonstrated, the OLP lesions improvement after viral eradication strongly supports a possible pathogenetic role of HCV chronic inflammation on OLP clinical course. These data could be crucial in the management of OLP, especially in countries where HCV is endemic such as in Italy.

## Declarations

### Research resource identifiers (RRID)

**Software:** ImageJ Version 1.53 k **RRID:SCR_003070**; IBM SPSS Statistics version 28.0.1.0 **RRID:SCR_019096**; G*Power version 3.1.9.6 RRID:SCR_013726.

## References

[1] Polaris Observatory HCV Collaborators (2017). Global prevalence and genotype distribution of hepatitis C virus infection in 2015: a modelling study. Lancet Gastroenterol Hepatol.

[2] Spearman CW, Dusheiko GM, Hellard M, Sonderup M (2019). Hepatitis C. Lancet.

[3] WHO/Europe | Fact sheet - Hepatitis C in the WHO European Region (2021). https://www.euro.who.int/en/health-topics/communicable-diseases/hepatitis/data-and-statistics/fact-sheet-hepatitis-c-in-the-who-european-region-2021. Accessed 1 Dec 2021

[4] Andriulli A, Stroffolini T, Mariano A (2018). Declining prevalence and increasing awareness of HCV infection in Italy: A population-based survey in five metropolitan areas. Eur J Intern Med.

[5] Marascio N, Liberto M, Barreca G (2014). Update on epidemiology of HCV in Italy: focus on the Calabria Region. BMC Infect Dis.

[6] Marrone A, Ciotti M, Rinaldi L (2020). Hepatitis B and C virus infection and risk of haematological malignancies. J Viral Hepatitis.

[7] Allison RD, Tong X, Moorman AC (2015). Increased incidence of cancer and cancer-related mortality among persons with chronic hepatitis C infection, 2006–2010. J Hepatol.

[8] Villanueva A (2019). Hepatocellular Carcinoma. N Engl J Med.

[9] Westbrook RH, Dusheiko G (2014). Natural history of hepatitis C. J Hepatol.

[10] Zampino R, Marrone A, Restivo L (2013). Chronic HCV infection and inflammation: Clinical impact on hepatic and extra-hepatic manifestations. World J Hepatol.

[11] Cacoub P, Saadoun D (2021). Extrahepatic Manifestations of Chronic HCV Infection. N Engl J Med.

[12] Marrone A, Di Bisceglie AM, Fox P (1995). Absence of hepatitis C viral infection among patients with primary Sjögren’s syndrome. J Hepatol.

[13] Ferri C, Sebastiani M, Giuggioli D (2015). Hepatitis C virus syndrome: A constellation of organ- and non-organ specific autoimmune disorders, B-cell non-Hodgkin’s lymphoma, and cancer. World J Hepatol.

[14] Di Stasio D, Mosca L, Lucchese A (2019). Salivary mir-27b Expression in Oral Lichen Planus Patients: A Series of Cases and a Narrative Review of Literature. Curr Top Med Chem.

[15] Alaizari NA, Al-Maweri SA, Al-Shamiri HM (2016). Hepatitis C virus infections in oral lichen planus: a systematic review and meta-analysis. Aust Dent J.

[16] Carrozzo M, Scally K (2014). Oral manifestations of hepatitis C virus infection. World J Gastroenterol.

[17] Carrozzo M, Quadri R, Latorre P (2002). Molecular evidence that the hepatitis C virus replicates in the oral mucosa. J Hepatol.

[18] Petta S, Adinolfi LE, Fracanzani AL (2018). Hepatitis C virus eradication by direct-acting antiviral agents improves carotid atherosclerosis in patients with severe liver fibrosis. J Hepatol.

[19] Adinolfi LE, Petta S, Fracanzani AL (2020). Impact of hepatitis C virus clearance by direct-acting antiviral treatment on the incidence of major cardiovascular events: A prospective multicentre study. Atherosclerosis.

[20] Persico M, Aglitti A, Caruso R (2018). Efficacy and safety of new direct antiviral agents in hepatitis C virus-infected patients with diffuse large B-cell non-Hodgkin’s lymphoma. Hepatology.

[21] Cacoub P, Desbois AC, Comarmond C, Saadoun D (2018). Impact of sustained virological response on the extrahepatic manifestations of chronic hepatitis C: a meta-analysis. Gut.

[22] Pawlotsky JM, Negro F, Aghemo A (2018). EASL Recommendations on Treatment of Hepatitis C 2018. J Hepatol.

[23] Erman A, Sathya A, Nam A (2018). Estimating chronic hepatitis C prognosis using transient elastography-based liver stiffness: A systematic review and meta-analysis. J Viral Hepat.

[24] Rueden CT, Schindelin J, Hiner MC (2017). Image J2: ImageJ for the next generation of scientific image data. BMC Bioinformatics.

[25] Piboonniyom S-O, Treister N, Pitiphat W, Woo S-B (2005). Scoring system for monitoring oral lichenoid lesions: a preliminary study. Oral Surg Oral Med Oral Pathol Oral Radiol Endod.

[26] Lavaee F, Shadmanpour M (2019). Comparison of the effect of photodynamic therapy and topical corticosteroid on oral lichen planus lesions. Oral Dis.

[27] Lauletta G, Russi S, Pavone F (2017). Direct-acting antiviral agents in the therapy of hepatitis C virus-related mixed cryoglobulinaemia: A single-centre experience. Arthritis Res Ther.

[28] Garcovich S, Garcovich M, Capizzi R (2015). Cutaneous manifestations of hepatitis C in the era of new antiviral agents. World J Hepatol.

[29] Bakhtiari S, Azari-Marhabi S, Mojahedi SM (2017). Comparing clinical effects of photodynamic therapy as a novel method with topical corticosteroid for treatment of Oral Lichen Planus. Photodiagn Photodyn Ther.

[30] Mester A, Lucaciu O, Ciobanu L (2018). Clinical features and management of oral lichen planus (OLP) with emphasis on the management of hepatitis c virus (HCV)-related OLP. Bosnian J Basic Med Sci.

[31] Scott GD, Rieger KE (2016). New-onset cutaneous lichen planus following therapy for hepatitis C with ledipasvir-sofosbuvir. J Cutan Pathol.

[32] Lodi G, Scully C, Carrozzo M (2005). Current controversies in oral lichen planus: report of an international consensus meeting. Part 1. Viral infections and etiopathogenesis. Oral Surg Oral Med Oral Pathol Oral Radiol Endod.

[33] Nagao Y, Kimura K, Kawahigashi Y, Sata M (2016). Successful Treatment of Hepatitis C Virus-associated Oral Lichen Planus by Interferon-free Therapy with Direct-acting Antivirals. Clin Transl Gastroenterol.

